# Primary Pericardial Well-Differentiated Papillary Mesothelioma in a Spotted Hyena (*Crocuta crocuta*)

**DOI:** 10.3390/vetsci12121170

**Published:** 2025-12-09

**Authors:** Louise van der Weyden, Dewald Keet, Nicolize O’Dell

**Affiliations:** 1 Wellcome Sanger Institute, Wellcome Genome Campus, Hinxton, Cambridge CB10 1SA, UK; lvdw@sanger.ac.uk; 2 Centre for Wildlife Health, Pretoria National Zoological Gardens, South Africa Biodiversity Institute, Pretoria 0001, South Africa; dewald.keet@up.ac.za; 3 Department of Veterinary Tropical Diseases, Faculty of Veterinary Science, University of Pretoria, Onderstepoort 0110, South Africa; 4 Department of Paraclinical Sciences, Faculty of Veterinary Science, University of Pretoria, Onderstepoort 0110, South Africa; 5 Centre for Veterinary Wildlife Studies, Faculty of Veterinary Science, University of Pretoria, Onderstepoort 0110, South Africa

**Keywords:** mesothelioma, papillary, PPM, pericardial, hydropericardium, heart failure, cardiogenic shock, hyena, hyaenidae

## Abstract

There have been few reports of neoplasia in hyenas to date. In this report, we describe a captive adult female spotted hyena (*Crocuta crocuta*) that developed inappetence, lethargy and marked abdominal distension over a 3-day period. The hyena was chemically immobilised to allow clinical investigation of the severe symptoms; however, she died before any internal examination occurred. At necropsy, severe serosanguinous hydropericardium was evident, as well as pulmonary congestion and oedema, ascites and chronic passive congestion of the liver with mild fibrosis. Histopathological examination of the pericardial surface revealed fibrous proliferations lined by mostly a single layer of large proliferating neoplastic mesothelial cells forming papillary projections into the lumen of the pericardial sac as well as infiltration into the pericardial connective tissue, with innumerable haemosiderin-laden macrophages in places, suggestive of chronic haemorrhage. The liver revealed severe congestion and interstitial fibrosis, and the lung revealed congestion and oedema, with moderate numbers of alveolar macrophages and marked anthracosis. The diagnosis was pericardial well-differentiated papillary mesothelioma, with death under anaesthesia caused by cardiogenic shock due to pericardial mesothelioma-associated cardiac tamponade. As primary pericardial mesothelioma (PPM) is a rare tumour type for both animals and humans, and this is the first report of a PPM in a hyena, we compare the clinical findings with those seen in other species.

## Simple Summary

There have been few reports of tumours in hyenas to date. In this report, we describe an adult female spotted hyena (*Crocuta crocuta*) that stopped eating, showed a reluctance to move and developed a distended abdomen over a 3-day period. The hyena was immobilised to allow clinical investigation; however, she died whilst under sedation. At necropsy, a severe accumulation of fluid in the sac surrounding the heart was noted, as well as fluid in the lungs, chest and abdominal cavity. There was also evidence of longstanding back damming of blood into the liver congestion. Histopathological examination of the surface of the heart revealed a single layer of large proliferating neoplastic mesothelial cells forming projections into the sac surrounding the heart, and macrophages with an appearance suggestive of chronic haemorrhage. The liver showed the presence of blood and fibrosis, and the lung also revealed a pleural effusion. The diagnosis was pericardial well-differentiated papillary mesothelioma, with death caused by heart failure induced by the tumour-associated pericardial effusion. As primary pericardial mesothelioma (PPM) is a rare tumour type, and this is the first report of a PPM in a hyena, we compare the clinical findings with those seen in other species.

## 1. Introduction

Primary pericardial mesothelioma (PPM) is a neoplasm arising from the pericardial mesothelial cell layers. PPM is exceptionally rare in humans, with an incidence of only 0.0022% [1], and accounting for 2–3% of primary heart and pericardial tumours [2]. PPM is also extremely rare in animals; dogs have the most reports of PPM [3,4,5,6,7], with only a single case report of PPM in a cat [8], a horse [9], and a Bengal tiger (*Panthera tigris*) [10]. In both humans and animals, the diagnosis is typically challenging, especially discerning this from papillary mesothelial hyperplasia (PMH), and is generally discovered late in a patient’s clinical course or at necropsy [11,12]. Usually, it is a highly aggressive malignancy, and, due to the typically late presentation, as well as an inability to remove via surgery, and poor response to chemotherapy/radiotherapy, the prognosis for PPM is extremely poor (with a pericardiectomy generally not curative). A well-differentiated slowly progressive variant classified as well-differentiated papillary mesothelioma (WDPM) is even rarer, with limited reports in the pericardium of humans and dogs [13,14,15]. Although still rare, this variant occurs more commonly in the peritoneum and pleura of humans and is often associated with endometriosis and asbestos exposure, respectively [14,16,17,18,19,20].

The Hyaenidae family is composed of four species: spotted hyena (*Crocuta crocuta*), brown hyena (*Hyaenea brunnea*), striped hyena (*Hyaena hyaena*) and aardwolf (*Proteles cristatus*). To date, there are only a handful of reports of tumours in Hyaenidae family members. A retrospective study of neoplasm necropsies at Taipei Zoo (Taiwan) during 1994–2003 reported a peritoneal mesothelioma in an aardwolf, a mammary gland carcinoma in a brown hyena and a lymphoma and bronchioloalveolar carcinoma in two striped hyenas [21]. Another study reported a population of wild spotted hyenas from the Masai Mara Game Reserve (Kenya) showing oral and genital cutaneous papillomas, from which a novel papillomavirus was detected [22]. Individual case reports include spotted hyenas with metastatic lymphosarcoma [23], chronic T-cell lymphocytic leukaemia [24] and anal sac squamous cell carcinoma [25]. In this report, we present the details of a case of PPM—more specifically, a well-differentiated papillary mesothelioma (WDPM) in a spotted hyena, and compare the findings with PPM, WDPM and PMH seen in other species.

## 2. Case Report

A captive adult (>16 years) intact female spotted hyena (*Crocuta crocuta*) from the South African National Zoological Gardens in Pretoria showed signs of inappetence and no urine or faeces was present in the enclosure over a 3-day period. The animal caretakers reported the hyena was depressed and lethargic and appeared bloated, with brown mucus membranes and halitosis. A tentative diagnosis of kidney failure or gastrointestinal obstruction was suspected. To allow investigation of the severe clinical signs, the hyena was immobilised (Zoletil 100; 350 mg; Virbac, Gauteng, South Africa) the following day. Whilst under anaesthesia, the hyena was administered a Ringer’s lactate drip, as well as an anti-spasmodic (Buscopan; 10 mL at 20 mg/mL; Boehringer Ingelheim Animal Health, Midrand, South Africa), an anti-inflammatory (Pyroflam; 1.75 mL at 50 mg/mL; Norbrook Laboratories, Gauteng, South Africa), a multi-nutritional supplement (Kyroligo; 7 mL of 100 mL/vial; Kyron Laboratories, Johannesburg, South Africa) and a vitamin B12 and phosphorus supplement (Catosal; 7 mL; 10% 100 mL/vial; Bayer, Midrand, South Africa); however, she died before any clinical examination could be performed. Blood was immediately collected and sent together with the body for clinical and histopathological analysis.

**Figure 1:**
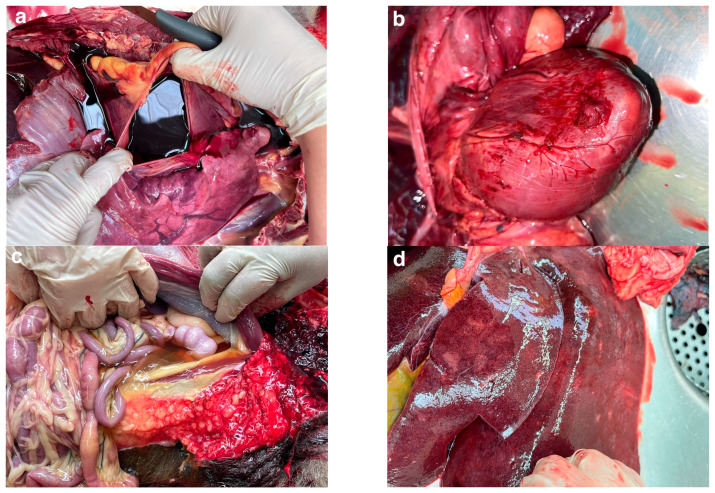
Macroscopic images of the spotted hyena autopsy. (**a**) Severe serosanguinous hydropericardium. (**b**) Numerous papillary projections and roughened areas on the visceral pericardium/epicardium. (**c**) Severe ascites upon opening the abdominal cavity. (**d**) Moderate chronic passive congestion of the liver with mild fibrosis.

Post-mortem laboratory tests found the blood smear results were within normal limits, and faecal flotation did not reveal the presence of any parasitic ova or oocysts. The body condition score was 3–3.5/5, with good muscle mass and adequate fat reserves. At necropsy, severe serosanguinous hydropericardium was evident (Figure 1a). Centrifuged impression smear of the hydropericardium fluid showed erythrocytes (4+), nests of abnormal mesothelial cells (2+), lymphocytes (2+), macrophages (2+) and non-degenerate neutrophils (2+), but no bacteria. The epicardial surface revealed numerous papillary projections and roughened areas that were blood-stained in areas (Figure 1b). Also noted was moderate-to-severe pulmonary congestion and oedema with moderate hydrothorax, moderate-to-severe ascites (Figure 1c) and moderate chronic passive congestion of the liver with mild fibrosis (Figure 1d). To aid the diagnosis, tissue samples from each of the organs were placed into 10% buffered formalin and submitted to the Histopathology Laboratory of the Faculty of Veterinary Science, University of Pretoria for routine histopathology, as previously described [26].

**Figure 2:**
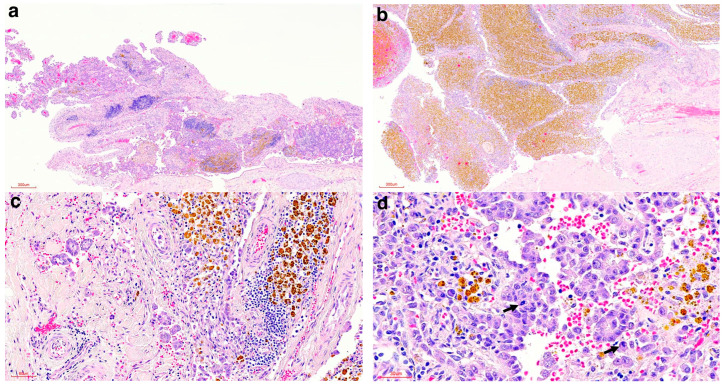
Histologic findings of the pericardial proliferations of the spotted hyena. (**a**) Proliferating neoplastic papillary projections (HE stain, 40× magnification). (**b**) Proliferating neoplastic papillary projections with marked accumulation of haemosiderin-laden macrophages (HE stain, 40× magnification). (**c**) Tubular structures infiltrating the underlying visceral pericardium (HE stain, 200× magnification). (**d**) Pleomorphic neoplastic mesothelial cells with mitoses (arrows) (HE stain, 400× magnification).

Examination of the haematoxylin and eosin (HE)-stained sections (4 μm) revealed the pericardial surface was characterised by fibrous proliferations lined by mostly a single layer of large proliferating neoplastic mesothelial cells forming papillary projections into the lumen of the pericardial sac (Figure 2a). One larger proliferative mass, situated at the base of the heart, consisted of the same mesothelial-lined papillary projections, together with innumerable haemosiderin-laden macrophages, suggestive of chronic haemorrhage (Figure 2b). In multiple areas associated with the papillary projections, the neoplastic mesothelial cells formed tubular structures infiltrating into the underlying connective tissue of the visceral pericardium (Figure 2c). The neoplastic cells revealed a moderate-to-severe degree of pleomorphism, with nuclear atypia and mitoses observed in most fields (Figure 2d). Apart from the papillary projections showing occasional mild mixed inflammation in their fibrous cores, the non-affected pericardium did not reveal evidence of inflammation. The liver was characterised by severe congestion, with mild-to-moderate interstitial fibrosis especially affecting the centrilobular areas, and the lung showed moderate congestion and oedema, with moderate numbers of alveolar macrophages and marked anthracosis. All other organs appeared within normal limits.

**Figure 3:**
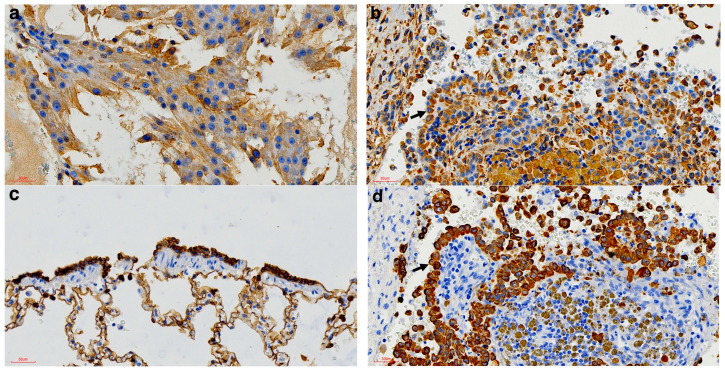
Immunohistochemical findings of the pericardial mass of the spotted hyena. (**a**) Positive Vimentin control from a Sertoli cell tumour (vimentin IHC, 400× magnification). (**b**) Vimentin-specific positive labelling of the neoplastic cells (arrow) (Vimentin IHC, 400× magnification). (**c**) Positive cytokeratin control from normal bronchiolar epithelium (Cytokeratin IHC, 400× magnification). (**d**) Cytokeratin-specific positive labelling of the neoplastic cells (arrow) (cytokeratin IHC, 400× magnification).

In addition, to confirm the neoplastic cells were of mesothelial origin and to exclude the differential diagnosis of a metastatic adenocarcinoma, immunohistochemical labelling was performed on the pericardial lesions. This was achieved by applying specific antibodies against vimentin and cytokeratin following the standard immunohistochemical protocols used at the Histopathology Laboratory, Faculty of Veterinary Science, University of Pretoria [27]. Mesothelial origin was confirmed with the positive labelling of the neoplastic cells by both vimentin (Figure 3a,b) and cytokeratin (Figure 3c,d).

The post-mortem investigation revealed a chronic active neoplastic process involving the mesothelial layer of the pericardium, and, considering the cellular morphology and growth pattern, it was consistent with a PPM, more specifically a WDPM. The pericardial effusion that accumulated around the heart resulted in external pressure on the heart, thus preventing it from contracting properly. This in turn resulted in secondary congestive heart failure and terminally cardiogenic shock. The severity and chronicity were evident by the changes that were observed in the lungs, liver and abdominal cavity, all indicating chronic congestion, due to the inability of the heart to contract properly. This condition is not conducive to life and irrespective of the immobilisation procedure it is unlikely the hyena would have survived.

## 3. Discussion

PPM is an extremely rare malignancy with a very poor prognosis in both humans and dogs. It typically poses a diagnostic challenge due to its late presentation, non-specific symptoms and diverse imaging manifestations, often making it a diagnosis of exclusion until cytological or histological samples are taken for analysis [28,29]. For example, in a study of three dogs that presented with recurrent pericardial effusions, ultrasound examination did not reveal any masses associated with the right atrium, aorta or other structures within the pericardial space, and an exploratory thoracotomy did not detect any neoplasms in all three cases [4]. A diagnosis was only able to be made after treatment with repeated pericardiocentesis and eventually by subtotal pericardiectomy, which allowed histopathological examination of the resected pericardial sacs that revealed nests of mesothelial cells [4]. Similarly, there are several case reports of human patients presenting with recurrent pericardial effusions, in which ultrasound, thoracic CT scans and/or analysis of the aspirated pericardial fluid were not able to detect any abnormalities, with the diagnosis of PPM coming from the histopathological analysis of a pericardial biopsy or the tissue removed via pericardiectomy [30,31,32]. Histopathological diagnosis of PPM can be aided with the use of immunohistochemical (IHC) staining. Consistent with the IHC results in this hyena, neoplastic cells staining positive for vimentin and cytokeratin have been used as a diagnostic aid for cases of PPM in humans [33], dogs [6,7], cats [34] and large felids [35], with positive vimentin staining typically excluding a diagnosis of metastatic adenocarcinoma [36].

An additional diagnostic challenge when diagnosing PPM is differentiating PPM, more specifically WDPM, from PMH. Reactive hyperplastic mesothelial cells can very easily be confused for neoplastic cells, and this pitfall is well-studied in the pleural and peritoneal cavity [19,20]; however, it has not been studied so much in the pericardial cavity [13,14,15]. In up to a third of cases, pathologists do not reach a consensus, and even more so when it is a well-differentiated mesothelioma. In almost all PMH cases, there are underlying pathological changes in the affected parts. In the peritoneal cavity, it is often associated with gynaecological disease such as endometriosis and asbestos exposure [16,20], while, in the pleura, asbestos exposure is a common aetiological factor [16,19], and in the pericardium it is usually associated with the inflammation or underlying myocardial and valvular lesions [37,38]. The only exception is cardiac mesothelial hyperplasia (CMH) in cattle, which may affect up to 90% of the pericardial surface [39]. At the most, in both humans and dogs, there may be mild nuclear atypia of the mesothelial cells and only rarely is there evidence of mitoses in PMH, and this is often similar in WDPM [20,37]. In cattle with CMH, the mesothelial cells may appear plump; however, dysplasia is minimal and invasion into the subjacent stroma is not a feature [39]. In our case, there was no evidence of an underlying condition. This, together with moderate cellular atypia, regular mitoses and multifocal areas of infiltration of the subjacent connective tissue, convinced us that these lesions were neoplastic in nature. It is, however, still important that one is aware of the fact that reactive mesothelial proliferations may resemble neoplastic lesions and that further diagnostic techniques should be pursued to arrive at the most accurate diagnosis [20]. One such technique to consider is the detection of BAP1 loss, since this is present in up to 60% of human mesotheliomas and is absent in non-neoplastic proliferative mesothelial lesions [40,41,42]. Unfortunately, 60% is also not absolute and this is still a largely unexplored technique for mesotheliomas in animals.

The presentation of PPM in this hyena has parallels with that seen in PPM of other species. For example, the development of a serosanguineous pericardial effusion has been reported in several human [43,44,45] and canine [4,5] PPM patients, as was also seen in this hyena. Pericardial effusion may occur in cases of severe PMH/CMH, but is generally not a common feature [39]. In humans, case reports of PPM have also included mention of constrictive pericarditis, cardiac tamponade and/or heart failure [11,12,46,47,48,49]; the latter two symptoms were also observed in this hyena. Consistent with the diagnosis of PPM being challenging, it is typically made late in the course of the disease or often after the patient has died. For example, a study of five canines with PPM reported that the diagnosis was only made at post-mortem examination [5]. Similarly, there are case reports of human patients in which a diagnosis was only made at autopsy [50,51]. This is in keeping with the course of the disease in this hyena, with a diagnosis only being made after death.

Currently, there is no consensus on treatment for PPM in humans or dogs. In a systematic review of the literature that included six case series and 84 case reports (encompassing 103 PPM cases) published from 2000 to 2016, multivariate analysis found that only chemotherapy was associated with improved survival, specifically chemotherapy with a platinum agent with or without pemetrexed [52]. In agreement with this, several recent case studies of human PPM patients treated with a combination of surgery and chemotherapy (carboplatin and pemetrexed) have reported the patient was still alive at the time of publication, which was 15–17-months post-treatment [53,54], with another case study using the same treatment protocol reporting that the patient had been through four cycles of chemotherapy with no complications and was in remission [55]. Similarly, in a retrospective multicentric study of 40 dogs with mesothelioma (including pleural, pericardial and peritoneal presentations), multivariate analysis found that chemotherapy was the only variable that was independently associated with survival (the chemotherapy regimens encompassed a variety of different treatment agents and protocols; however, most cases received intracavitary cisplatin and carboplatin) [56,57]. When considering only PPM, dogs treated with chemotherapy and/or surgery tended to live longer, but this was not statistically significant [56,57]. Primary cultures and xenograft models of canine PPM have shown that carboplatin is highly effective when used as a sole agent and in combination with gemcitabine [58]. A case study of a Yorkshire Terrier with PPM treated with surgery and intrathoracic carboplatin and gemcitabine reported survival for 7 months post-surgery, before dying of progressive respiratory distress [59]. More recently, a case study of a Golden Retriever with PPM that was treated with surgery and epirubicin highlighted the potential application of this drug; however, it stated that future studies were strongly recommended, to be able to conclude whether epirubicin effectively alleviated cardiac burden and achieved effective management of PPM [6].

There is also currently no consensus on the second line of treatment for PPM after platinum- and pemetrexed-based chemotherapy. Trials using immune checkpoint inhibitors as second line single agents and/or as part of multi-agent treatment regimes in unresectable malignant mesothelioma have shown promising results [57,60]. Although these trials have been for patients with pleural mesothelioma, there is a case report suggesting that some patients with advanced PPM may benefit from immune checkpoint inhibitors post platinum- and pemetrexed-based chemotherapy [60].

The aetiology of PPM is currently unknown. In contrast to pleural and peritoneal mesothelioma, the relationship between asbestos exposure and PPM is controversial; a literature review of PPM cases from 1993 to 2008 revealed only 3/14 had confirmed exposure to asbestos [61]. Other suspected risk factors for mesothelioma include exposure to the carcinogenic mineral fibre erionite, simian virus 40 infection and radiation exposure [62]; however, it is not clear whether these are specifically relevant for PPM. A study of five Golden Retrievers with a long-term history of recurrent idiopathic haemorrhagic pericardial effusion (IHPE) reported that diagnostic imaging, cytology and a microbiological culture of fluid obtained during repeated pericardiocentesis revealed no abnormalities, and, in the three dogs that eventually underwent a pericardiectomy, neoplastic lesions were not detected in any organs or tissues within the thoracic cavity during the surgical procedure or in the surgical biopsies [5]. However, PPM was diagnosed at post-mortem examination in all five dogs, with the clinical course of the disease ranging from 30 to 54 months (from first visit to death), and it was suggested that the recurrent IHPE caused chronic inflammatory processes that led to the development of PPM [5]. Consistent with this, in a case report of a 22-year-old male who underwent pericardiocentesis due to recurrent pericarditis and a large pericardial effusion, histopathology of the pericardium only revealed benign mesothelial inflammation, consistent with acute pericarditis [63]. However, four months later, the patient presented with a large pericardial mass manifesting in heart failure and histopathology of the pericardiectomy specimen revealed PPM, with the patient dying 3 weeks later [63]. Pathologically confirmed pericarditis transforming into PPM has also been reported in a 64-year-old female [64]. In our case, there was no evidence of a predisposing aetiology, such as asbestos exposure or chronic inflammation, and therefore we concluded that it was spontaneous development of the neoplastic lesion, which is not unheard of in other species where no causative or epizootiological factors could be identified [3].

The only evidence of any sort of environmental exposure in the tissues of this hyena was the anthracosis that was observed in the lung, which is not associated with mesothelioma, and is therefore an incidental finding in this case. Interestingly, the South African National Zoological Gardens were established in 1899 and are currently situated close to the city centre [65]. Due to this, the animals are continuously exposed to carbon in the air and anthracosis is a common finding in city-dwelling humans and animals, especially zoo animals, exposed to air pollution [66].

## 4. Conclusions

This case report of a hyena with PPM adds to our limited knowledge of this very rare tumour type and highlights the strengths of cross-species comparisons, in line with the One Medicine approach. In addition, given that many zoos, wildlife parks and/or game reserves across the globe are home to populations of Hyaenidae family members, it is important to document the different tumours that occur and detail the clinical and histopathological findings, to better aid veterinarians in their care of these animals.

## Data Availability

The original contributions presented in this study are included in the article. Further inquiries can be directed to the corresponding author.

## Abbreviations

The following abbreviations are used in this manuscript:

CMH	Cardiac mesothelial hyperplasia
HE	Haematoxylin and eosin
IHC	Immunohistochemistry
IHPE	Idiopathic haemorrhagic pericardial effusion
PMH	Papillary mesothelial hyperplasia
PPM	Primary pericardial mesothelioma
WDPM	Well-differentiated papillary mesothelioma
